# Effect of NIR laser therapy by MLS-MiS source against neuropathic pain in rats: *in vivo* and *ex vivo* analysis

**DOI:** 10.1038/s41598-019-45469-5

**Published:** 2019-06-26

**Authors:** Laura Micheli, Francesca Cialdai, Alessandra Pacini, Jacopo Junio Valerio Branca, Lucia Morbidelli, Valerio Ciccone, Elena Lucarini, Carla Ghelardini, Monica Monici, Lorenzo Di Cesare Mannelli

**Affiliations:** 1 Department of Neuroscience, Psychology, Drug Research and Child Health - NEUROFARBA - Pharmacology and Toxicology Section, University of Florence, Florence, Italy; 2 ASAcampus Joint Laboratory, ASA Res. Div. – Department of Experimental and Clinical Biomedical Sciences “Mario Serio”, University of Florence, Florence, Italy; 3 Department of Experimental and Clinical Medicine, University of Florence, Florence, Italy; 4 Department of Life Sciences, University of Siena, Siena, Italy

**Keywords:** Chronic pain, Trauma

## Abstract

Neuropathic pain is characterized by an uncertain etiology and by a poor response to common therapies. The ineffectiveness and the frequent side effects of the drugs used to counteract neuropathic pain call for the discovery of new therapeutic strategies. Laser therapy proved to be effective for reducing pain sensitivity thus improving the quality of life. However, its application parameters and efficacy in chronic pain must be further analyzed. We investigated the pain relieving and protective effect of Photobiomodulation Therapy in a rat model of compressive mononeuropathy induced by Chronic Constriction Injury of the sciatic nerve (CCI). Laser (MLS-MiS) applications started 7 days after surgery and were performed ten times over a three week period showing a reduction in mechanical hypersensitivity and spontaneous pain that started from the first laser treatment until the end of the experiment. The *ex vivo* analysis highlighted the protective role of laser through the myelin sheath recovery in the sciatic nerve, inhibition of iNOS expression and enhancement of EAAT-2 levels in the spinal cord. In conclusion, this study supports laser treatment as a future therapeutic strategy in patients suffering from neuropathic pain induced by trauma.

## Introduction

Neuropathic pain is the result of damage (due to injury or disease) to the nervous system (including nerves), spinal cord and other central nervous system regions^1–3^. Neuropathy patients suffer from spontaneous pain, allodynia (pain response to normally innocuous stimuli) and hyperalgesia (aggravated pain evoked by noxious stimuli) that interferes with their quality of life^4–6^. Several experimental models have been developed to better understand neuropathy. The chronic constriction injury (CCI) model, developed by Bennett and Xie^7^, is a widely used model of mononeuropathy that replicates in rats most of the symptoms occurring in patients^7–10^.

Currently the most common way to treat pain is the administration of pain relief medications, although they have proved to be effective in only 30% of neuropathy patients which makes the research for new and effective treatments an ongoing challenge^11–13^.

The history of investigation and clinical use of laser therapy in medicine goes back to the late 1960s^14^. Since then, laser irradiation has been acknowledged as one of the most important non-pharmacological therapies. Nowadays laser therapy use has become increasingly widespread because it is a non-invasive approach with few contraindications, rare side effects and relatively low costs, thus well accepted by patients^15,16^. The literature on laser therapy action mechanisms is extremely wide although with controversial findings that are difficult to compare and interpret, due to the very different experimental conditions (in particular the type of laser source and treatment parameters, that are wavelength, power, fluence, exposure time, etc…) used in the studies. However, through the years laser therapy has been has demonstrated its effectiveness in treating a number of different pathological conditions^17–24^. The possibility to apply laser therapy in so many different pathological states depends on the effects that radiation has on important biological processes.

A number of studies in literature have reported that laser radiation is effective in improving cell energy metabolism through ATP synthesis increase^25–27^. Further insights into the action mechanisms underlying enhanced cell energy metabolism were provided in a proteomic study, carried out on myoblasts exposed to near infrared (NIR) laser radiation (808 and 905 nm), where an increase in ATP-binding proteins and Protein Phosphatase 1 (PP1) was observed^28^. The same study, showed that laser irradiation induced a significant increase in NLRP10, an anti-inflammatory protein that inhibits the production of interleukin 1β^28^. The analgesic effect is also due to other mechanisms acting on the production of anti-nociceptive substances (endorphins), peripheral nerve conduction and the transmission of nociceptive stimuli, as demonstrated by the rapid analgesic effect evoked by laser radiation in animal models of persistent pain^18,29–31^.

Preliminary studies showed that the anti-hypersensitivity effect and its persistence depended on treatment protocols and parameters (irradiation mode, treatment frequency, source wavelength and power, pulse frequency)^31^.

The aim of the present study was to investigate the effectiveness of a high power, dual wavelength NIR laser source (Multiwavelength Locked System laser, MLS-MiS) in producing a persistent anti-hypersensitivity effect in CCI-induced neuropathy caused by compressive damage in the rat. The laser therapy action mechanism were also assessed with *ex vivo* evaluations of the central and the peripheral nervous system aimed to highlight the regeneration of the sciatic nerve and the reduction of the inflammatory processes in the spinal cord.

## Results

### Effect of laser treatments on CCI-induced hypersensitivity

**Figure 1:**
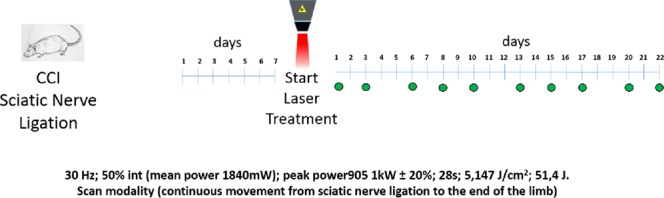
Laser treatment protocol with time schedule and parameters used.

**Figure 2:**
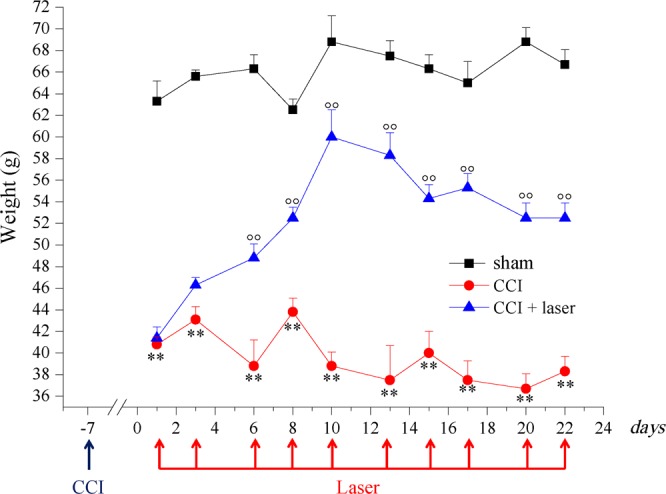
Monolateral neuropathy model induced by CCI. Sciatic nerve ligation was performed 7 days before the beginning of the test (day −7). Laser treatment [28 s, 30 Hz; 50% int (mean power 1840 mW); peak power_905_ 1 kW ± 20%; 5,147 J/cm^2^; 51,4 J] was applied on days 1; 3; 6; 8; 10; 11; 13; 15; 17; 20; 22 at 0 min and 30 min after laser application. The response to a noxious mechanical stimulus was measured by Paw pressure test. Values reported in the graph are referred to measurements conducted before treatments. Each value represents the mean ± S.E.M of 6 rats per group performed in two different experimental sets. **P < 0.01 vs sham group; °°P < 0.01 vs CCI group.

Behavioural measurements were performed to evaluate the anti-hypersensitivity effect of repeated laser treatments on CCI-induced peripheral mononeuropathy in the rat. Laser treatment started one week after surgery and consisted of 10 sessions every other day, until the 3^rd^ week (Fig. 1). The evaluation of hypersensitivity (Paw pressure test) was performed immediately before and 30 min after each laser application. Figure 2 shows the mean values monitored in the 3 groups of animals (sham, CCI, CCI + laser) before each of the 10 laser sessions.

The measurement performed before the first laser application (day 1) demonstrated that sciatic nerve ligation decrease the response to a mechanical noxious stimulus (Paw pressure test) from a value of 63.3 ± 1.9 g (sham animals group) to 40.8 ± 0.7 g (CCI and CCI + laser groups). In the CCI group this condition lingered for 3 weeks, until the end of the experiment (day 22). On the contrary, in the CCI + laser group two laser applications were enough to significantly increase the weight tolerated on the ipsilateral paw compared to the untreated CCI animals (48.8 ± 1.3 g and 38.8 ± 1.4 g, respectively, on day 6). The higher anti-hypersensitivity effect was recorded after four laser applications (day 10), with a value of 60.0 ± 2.5 g on the ipsilateral paw. Subsequent laser applications did not increase the paw threshold that remained stable (about 55 g) until the end of the experiment (day 22). In each laser session, the measurement performed 30 min after laser irradiation did not show any significant change in comparison to the pre-irradiation measurement (Table 1).

**Table 1:** Response to a mechanical noxious stimulus of CCI + laser treated animals, Paw pressure test.

Laser applications	Weight (g)	
	before treatment	after treatment
	0 min	30 min
1	41.8 ± 0.6**	49.6 ± 2.6
2	46.3 ± 0.7**	50.3 ± 3.6
3	48.8 ± 1.3*	50.0 ± 1.0
4	52.5 ± 1.0°°	51.3 ± 1.2
5	60.0 ± 2.5°°	58.3 ± 2.9
6	58.3 ± 2.1°°	55.0 ± 2.5
7	54.3 ± 1.3°°	52.5 ± 2.5
8	55.3 ± 1.3°°	52.5 ± 1.4
9	52.5 ± 1.4°°	53.8 ± 2.4
10	52.5 ± 1.4°°	56.3 ± 1.3

Paw pressure test performed on CCI + laser treated animals, 30 min after the daily laser treatment. Each value represents the mean ± s.e.m of 6 rats performed in two different experimental sets.

**Figure 3:**
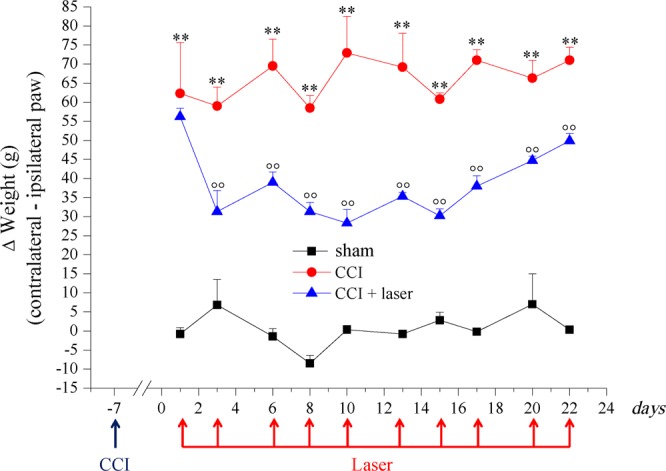
Monolateral neuropathy model induced by CCI. Sciatic nerve ligation was performed 7 days before the beginning of the test (day −7). Laser treatment [28 s, 30 Hz; 50% int (mean power 1840 mW); peak power_905_ 1 kW ± 20%; 5,147 J/cm^2^; 51,4 J] was applied on days 1; 3; 6; 8; 10; 11; 13; 15; 17; 20; 22 at 0 min and 30 min after laser application. The hind limb weight bearing alteration was measured by Incapacitance test. Values reported in the graph are referred to measurements conducted before treatments. Each value represents the mean ± S.E.M of 6 rats per group performed in two different experimental sets. **P < 0.01 vs sham group; °°P < 0.01 vs CCI group.

Monolateral pain as CCI induced alteration of hind limb weight bearing has been shown by the Incapacitance test (Fig. 3). Also in this case, measurements were performed immediately before each laser application and 30 min later. The values monitored before each laser session are reported in Fig. 3. Before the first laser treatment (day 1, 7 days after surgery), the difference between the weight burdened on the contralateral and the ipsilateral paw (Δ g) was significantly increased in both CCI and CCI + laser groups (about 60 g) compared to the sham group (−0.8 ± 1.7 g). In the CCI group this difference remained constant until the end of the experiment (day 22), while in the CCI + laser group the gap has been reduced by about 50% on the first laser application (31.3 ± 5.5 g, day 3). The pain relieving effect remained stable throughout the following laser treatments (days 6–16) to then slightly decrease from day 20. The measurements performed 30 min after each laser session did not show significant changes compared to the pre-irradiation measurements (Table 2).

**Table 2:** Hind limb weight bearing alterations of CCI + laser treated animals, Incapacitance test.

Laser applications	Δ Weight (g)	
	before treatment	after treatment
	0 min	30 min
1	56.2 ± 2.3**	47.9 ± 5.0
2	31.3 ± 5.5°°	27.9 ± 5.0
3	39.0 ± 2.7°°	33.3 ± 4.6
4	31.3 ± 2.4°°	30.3 ± 7.0
5	28.3 ± 3.6°°	33.9 ± 3.9
6	35.3 ± 1.0°°	32.5 ± 1.5
7	30.2 ± 1.8°°	35.8 ± 2.5
8	38.0 ± 2.7°°	47.8 ± 1.7
9	44.7 ± 1.2°°	48.4 ± 3.1
10	49.9 ± 1.9°°	51.2 ± 0.7

Incapacitance test performed on CCI + laser treated animals, 30 min after the daily laser treatment. Each value represents the mean ± s.e.m of 6 rats performed in two different experimental sets.

### Effect of laser treatments on CCI-induced sciatic nerve damage: histological evaluation

**Figure 4:**
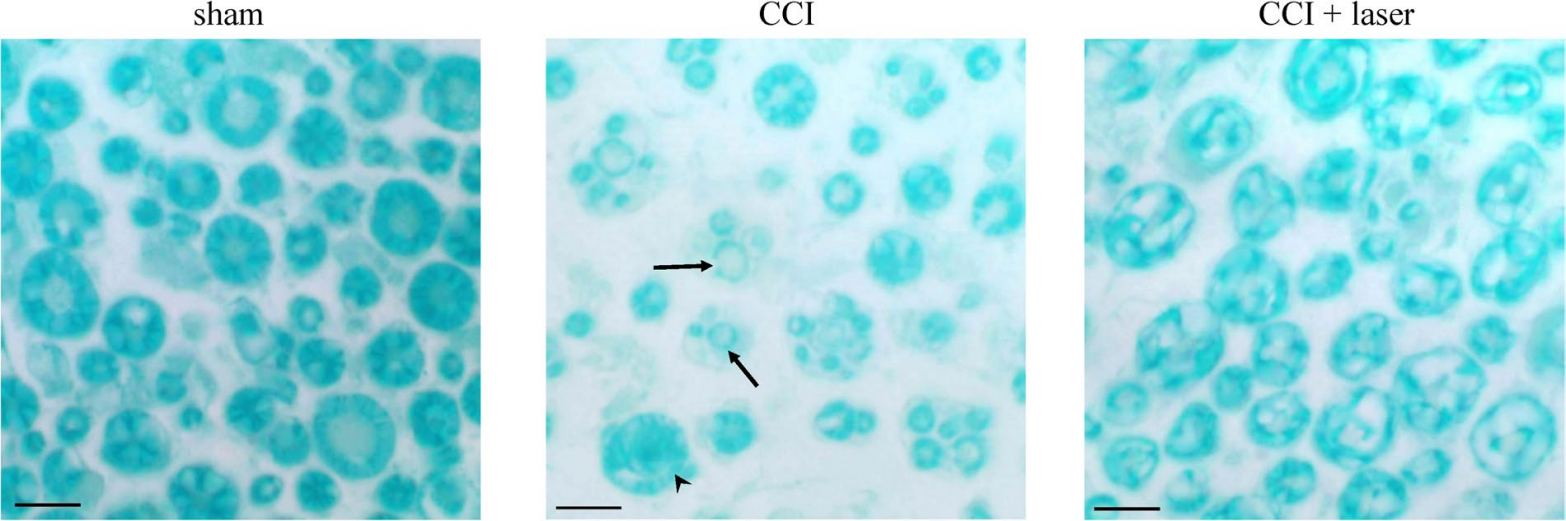
Luxol Fast Blue staining. Representative micrographs of sciatic nerve axons in a sham, CCI and CCI + laser groups showing a partial laser-dependent neuroprotection of myelin thickness. Original magnification 400 X. Scale bar = 20 μm.

The morphometric assessment by Luxol Fast Blue (LFB) tissue staining allowed to characterize the laser-dependent effect in comparison to the CCI-dependent alteration in the myelin sheath thickness. The histological examination of the specimens revealed a normal sciatic nerve appearance in the sham group with a regular distribution of small and large diameter nerve fibers as well as a normal proportion between myelin sheath thickness and fiber diameter (Fig. 4, sham). As expected, the CCI group presented a wide distribution of very thinly myelinated nerve fibers (Fig. 4, CCI – black arrows), Wallerian degeneration (Fig. 4, CCI – black arrowhead) and unmyelinated fibers in the tissue sections of the sciatic nerve 900 μm proximal to the ligation site, compared to sham rats. In contrast, nerves of animals treated with laser radiation (CCI + laser treated group) showed a remarkable myelin regeneration, as demonstrated by the presence of a greater number of nerve fibers that were surrounded by much more myelin compared with the CCI group (Fig. 4, CCI + laser). Table 3 shows the measurements of fiber and axonal diameters and myelin thickness. Laser treatment significantly increased (°°P < 0.01 vs CCI) the myelin thickness in comparison to CCI animals (2.24 ± 0.18 *vs* 1.81 ± 0.20) whereas fiber and axonal diameter measurements did not reveal any significant laser-dependent improvement.

**Table 3:** Morphometric analysis of sciatic nerves.

	Fiber diameter	Axonal diameter	Myelin thickness
sham	7.71 ± 0.32	3.17 ± 0.05	2.27 ± 0.16
CCI	6.33 ± 0.68**	2.72 ± 0.30**	1.81 ± 0.20**
CCI + laser	6.61 ± 0.49**	2.13 ± 0.18**	2.24 ± 0.18**°°

Morphometric analysis of sciatic nerves 29 days post-surgery showing the measurement of fiber and axonal diameter and of myelin thickness. Five µm sections stained with LFB were photographed at 100X magnification. The myelin thickness in photomicrographs taken from randomly selected fields was counted (6 rats/group) and analyzed using Origin 9.0 statistical software. The results showed a significant decrease in axonal and fiber diameter as well as in myelin thickness in CCI compared to sham group (**P < 0.01). Laser-treated group showed a significant increase in myelin thickness compared to CCI group (°°*P* < 0.01).

**Figure 5:**
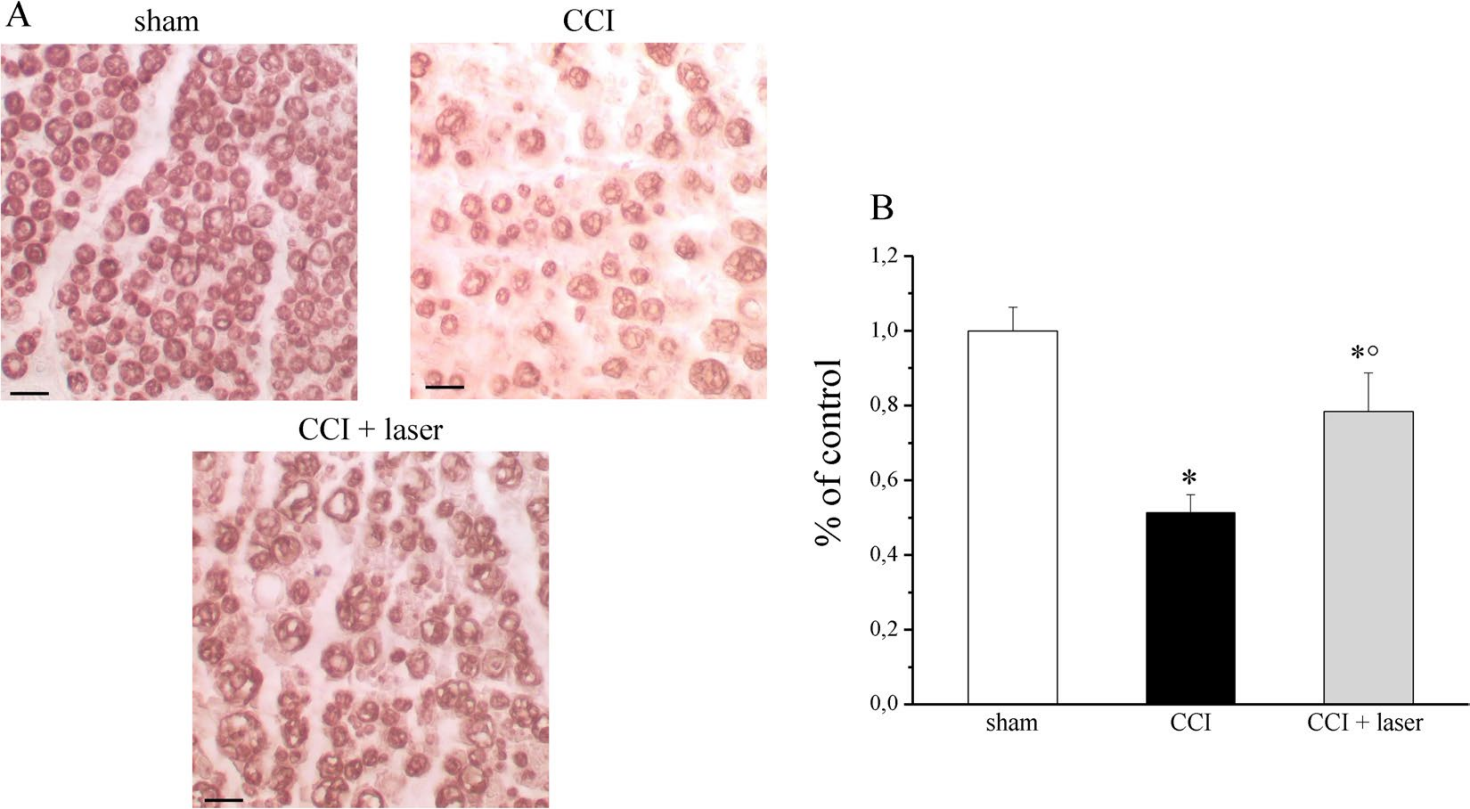
Myelin Basic Protein (MBP) expression. A, Protein expression of MBP was evaluated by immunohystochemistry in each experimental group. CCI group and CCI + laser group were compared to each other and with sham group. Original magnification 400X. Scale bar = 20 μm. B, AEC intensity was calculated by the integrated density of pixels for MBP. Control condition was arbitrarily set as 100% and results are expressed as mean ± S.E.M of 6 rats per group. Results are representative of at least three independent immunohistochemistry evaluations. *P < 0.05 vs sham group and °P < 0.01 vs CCI group. Each experimental point was performed in triplicate. Pictures are representative of fifteen field captured for each experimental point. *P < 0.01 vs sham group; °P < 0.01 vs CCI group.

Based on the results described above, to further investigate the effectiveness of NIR laser radiation in nerve protection and myelin sheath regeneration, Myelin Basic Protein (MBP) expression was evaluated by immunocytochemistry (Fig. 5). MBP is a major constituent of the myelin sheath produced by Schwann cells in the peripheral nervous system. On day 22 (end of laser treatments, 30 days post-injury), MBP was significantly lower in the CCI group compared to the sham one (*P < 0.01 vs sham). Laser treatment partially restored the MBP in the sciatic nerve of CCI + laser group (°P < 0.01 vs CCI). These data further confirm the laser-dependent neuroprotection, in particular the restoration of the myelin sheet, revealed by morphometric analysis.

### Effect of laser treatments on inflammatory markers

**Figure 6:**
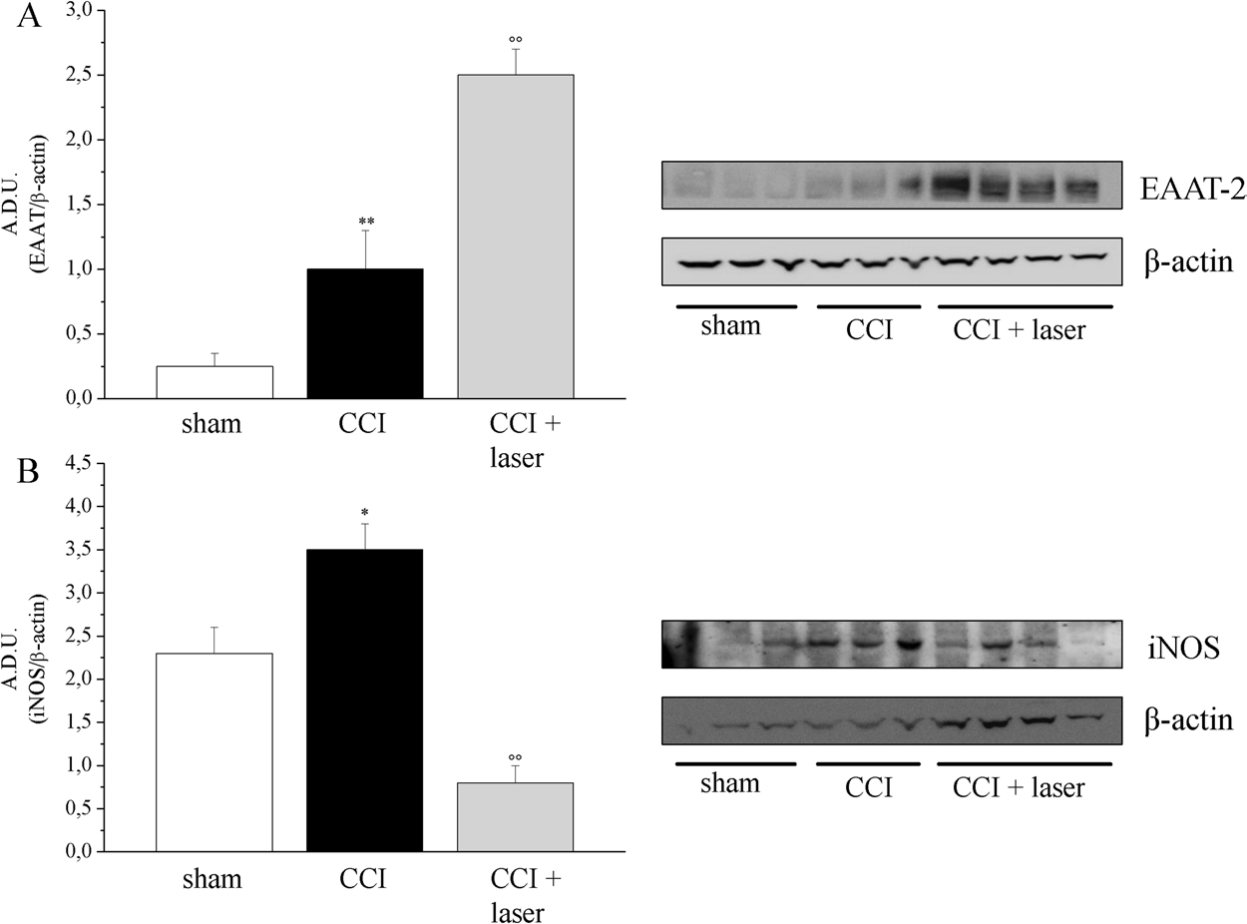
Western blot analysis of inflammatory markers. Spinal cords were isolated at the end of the experiment and proteins were run on SDS-PAGE. Proteins transferred on nitrocellulose membranes were then labelled with primary antibodies against EAAT-2 (panel A) and iNOS (panel B). β-actin normalization was performed f each sample. The graphs represent the means ± S.E.M of 6 rats per group. *P < 0.05 and **P < 0.01 vs sham group; °°P < 0.01 vs CCI group.

On day 22 (end of laser applications, 30 days post-injury), the nervous tissue (spinal cord) was evaluated for the expression of the glutamic acid transporter EAAT-2 and the inflammatory markers iNOS, COX-2 and mPGES-1. Results showed that while EAAT-2 was scarcely expressed in the spinal cord of sham animals, the CCI group (laser-untreated) presented an increased expression, that was even higher in laser treated animals (CCI + laser group) (Fig. 6A).

A panel of inflammatory markers was then evaluated as the inducible isoform of NOS (iNOS) and the prostanoid pathway key enzymes COX-2 and mPGES-1. While COX-2 and mPGES-1 were not detected under any experimental condition (data not shown), the CCI procedure induced an increase in iNOS expression, whose levels were significantly blunted by laser application, with value even below the sham group (Fig. 6B).

## Discussion

Our previous studies showed the effectiveness of NIR laser therapy in reducing CCI-induced pain in the rat^32^. A remarkable analgesic effect, whose peaked at about 30 min after laser treatment, was obtained with a protocol consisting in the irradiation of two points, the first one directly located on sciatic nerve ligation and the other one on lateral side of the calcaneus (paw joint). However, the analgesia decreased rapidly (about 2 hours later). In further studies, a more persistent analgesic effect was achieved when the irradiation of the two fixed points was followed by a scan on the whole leg, but even in this case the effect vanished 24 hours later^31^.

Based on these evidences, in the present study a new protocol was designed and tested to obtain a long-lasting anti-hypersensitivity and protective effect through anti-inflammatory action and repair mechanisms. Results showed that laser treatment, performed by scans of the entire leg with the synchronized emission of a NIR, dual wavelength, high power source (MLS-MiS), was able to control pain and inhibit the progression of a persistent painful condition. The loose ligation of the sciatic nerve induces a damage characterized by painful sensations correlated with overt tissue alterations. As previously reported, CCI model elicits a pain syndrome characterized by mechanical and thermal hyperalgesia that begins about 3 days after nerve injury and reaches a plateau from 7 up to 30 days^33^. Laser treatment over these 3 weeks (10 sessions, every other day) counteracted the development of mechanical hyperalgesia already after two applications. The maximum anti-hyperalgesic effectiveness was reached with 5 sessions, then the effect remained more or less stable until the end of the experiment. It is noteworthy that the measurements performed 30 min after laser irradiation did not show significant differences compared to the pre-treatment ones. While, over the first 5 sessions, an evident increase in the pain threshold was recorded between each before-irradiation measurement and the measurements performed before and after the previous laser session (48 h before). This means that the protocol used in this study does not induce an immediate analgesic effect, but rather a biological response rising more slowly but lasting longer over time.

The treatment protocol applied in this study was also able to reduce postural unbalance, a feature of neuropathy progression measured by hind limb weight bearing alterations. This measurement, in particular, may assess the somatosensory component of mononeuropathy highlighting spontaneous non evoked pain^34^. Laser treated animals showed a halving of postural unbalance measurement from the first treatment and this effect remained constant until the end of the laser sessions, as the Incapacitance test showed. Also in this case, the measurements performed 30 min after each laser irradiation did not show significant differences compared to the pre-treatment ones, confirming the paw pressure test results. The *ex vivo* analysis also highlighted a protective role of laser treatment in the central and peripheral nervous system.

In agreement with previous results^33,35,36^, histology, immunohistochemistry and levels of inflammatory markers (evaluated by western blot) showed that CCI-induced morphometric alterations of the sciatic nerve that dramatically affect the proximal distal from the injury. Besides, CCI mediated nerve architecture derangement is accompanied by local inflammatory reaction response, which include oedema, infiltration of hematogenous immune cells and induction of various soluble factors like cytokines, chemokines and small signalling molecules as nitric oxide. These findings were further confirmed by the increased expression of iNOS detected in spinal cord samples. Laser treatment significantly prevented the reduction in myelin sheath thickness and hindered myelin degeneration, as highlighted by LFB staining and MBP immunohistochemistry. This result is in agreement with data reported by other authors, showing that NIR laser therapy was able to promote nerve fiber regeneration and improve the quality of myelin layers in a rabbit model of peripheral nerve injury^37^. Moreover, previous data obtained using sources with emission 808 nm and 904 nm, the same wavelengths used in the present study, demonstrated that these NIR radiations enhanced nerve repair after end-to-side neurorrhaphy of the median nerve in a rat model^38^ and increased HNRNPK expression in cell culture^28^. HNRNPK is a member of the heterogeneous nuclear ribonucleoproteins (hnRNPs) subfamily known to be required for axonogenesis during development and several of its RNA targets are under strong post-transcriptional control during the regeneration process^39^.

The anti-inflammatory effect elicited by laser application in the CCI model was clearly demonstrated by the significant reduction in iNOS expression in the spinal cord. Also this result is consistent with previous studies^28^ that highlighted the increase of NLRP10 protein, an inflammasome inhibitor induced by laser radiation with 808 nm and 904 nm wavelengths.

In the central nervous system, the excitatory amino acid transporters (EAATs) remove glutamate from the synaptic cleft and extrasynaptic sites via glutamate reuptake into glial cells and neurons, allowing to keep its levels low and to terminate the synaptic transmission. Conditions that increase the levels of EAAT-2 expression, may avoid an excess of glutamate capable of triggering a series of biochemical cascades associated to excitotoxicity and neuronal damage^40^. The findings of this study show that repeated laser treatments were able to strongly increase EAAT-2 levels, corroborating the anti-inflammatory and beneficial effects of the therapy on nervous and glial cells.

In conclusion, the results of this study indicate that NIR laser therapy carried out with MLS-MiS laser source and suitable protocols is able to control pain and prevent alterations of the nervous system induced by nerve injury.

While our previous studies^31^, reported a fast but not lasting analgesic effect was obtained with point by point irradiation, the protocol used in this study activated a slower but longer lasting biological response that counteracted hyperalgesia through three different mechanisms: (1) anti-inflammarory effect via inhibition of iNOS expression; (2) repair effect through preservation/restoration of myelin sheath; (3) protective effect on central nervous system via enhancement of EAAT-2 levels. The collected data present a preclinical evaluation for a future therapeutic application of laser in patients suffering from neuropathic pain induced by trauma. The protocols can be further studied in order to exploit both the rapid analgesic effect that can be obtained by trigger point irradiation and the more persistent protective effect, with direct action on the cause of pain.

## Materials and Methods

### Animals

In all the experiments described below, male Sprague–Dawley rats (Envigo, Varese, Italy) weighing approximately 200–250 g at the beginning of the experimental procedure were used. Animals were housed in a Laboratory Animal Facility (CeSAL, Centro Stabulazione Animali da Laboratorio, University of Florence) and used one week after their arrival. Four rats were housed per cage (size 26 × 41 cm^2^), fed with standard laboratory diet and tap water ad libitum, kept at 23 ± 1 °C with a 12 h light/dark cycle, light at 7 a.m. All animal manipulations were carried out according to the Directive 2010/63/EU of the European Parliament and of the European Union council (22 September 2010) on the protection of animals used for scientific purposes. The ethical policy of the University of Florence complies with the Guide for the Care and Use of Laboratory Animals of the US National Institutes of Health (NIH Publication No. 85–23, revised 1996; University of Florence assurance number: A5278-01). Formal approval to conduct the experiments described was obtained from the Italian Ministry of Health (No. 54/2014-B) and from the Animal Subjects Review Board of the University of Florence. Experiments involving animals have been reported according to ARRIVE guidelines^41^. All efforts were made to minimize animal suffering and to reduce the number of animals used.

### CCI-induced peripheral mononeuropathy

Neuropathy was induced according to the procedure described by^7^. Briefly, rats were anaesthetized with 2% isoflurane. Under aseptic conditions, the right (ipsilateral) common sciatic nerve was exposed by blunt dissection at the level of the mid thigh. Proximal to the trifurcation, the nerve was carefully freed of the adhering tissue from the surrounding connective tissue, and 4 chromic catgut ligatures (4-0, Ethicon, Norderstedt, Germany) were tied loosely around the nerve with about 1-mm spacing between ligatures. After haemostasis was confirmed, the incision was closed in layers. The animals were allowed to recover from surgery and then housed one per cage with free access to water and standard laboratory chow. Another group of rats were subjected to sham surgery in which the sciatic nerve was only exposed but not ligated. Laser treatment started 7 days after surgery.

### Laser treatment and study design

Treatment were performed with a Multiwave Locked System laser (MLS-MiS, ASA S.r.l., Vicenza, Italy), a class IV NIR laser with two synchronized sources (laser diodes): the first one is a pulsed laser diode emitting at 905 nm wavelength, with peak power from 140 W ± 20% to 1 kW ± 20% and pulse frequency varying in the range 1–2000 Hz; the second laser diode emitting at 808 nm wavelength can operate in continuous (max power 6 W ± 20%) or frequenced (repetition rate 1–2000 Hz, 50% duty cycle) mode. The two laser beams work simultaneously, synchronously and the propagation axes are coincident.

Seven days after the sciatic nerve ligation, animals were randomly distributed into three groups:

- **Sham** (n = 6), animals subjected to sham surgery in which the sciatic nerve was only exposed but not ligated.
- **CCI** (n = 6), animals subjected to the ligation of sciatic nerve, untreated with laser;
- **CCI** + l**aser** (n = 6), animals subjected to the ligation of sciatic nerve, treated with laser. The treatment was performed 3 times a week over a 3 week period (days 1; 3; 6; 8; 10; 11; 13; 15; 17; 20; 22) for a total of 10 applications (Fig. 1) and consisted in a limb scan with the handpiece constantly moved over the treatment area. Irradiation was performed for 28 s with the following parameters: 30 Hz; 50% int (mean power 1840 mW); peak power_905_ 1 kW ± 20%; 5,147 J/cm^2^; 51,4 J.

### Paw pressure test

The nociceptive threshold in the rat was determined with an analgesimeter (Ugo Basile, Varese, Italy) according to the method described by^42^. Briefly, a constantly increasing pressure was applied to a small area of the dorsal surface of the hind paw using a blunt conical mechanical probe. Mechanical pressure was increased until vocalization or a withdrawal reflex occurred while rats were lightly restrained. Vocalization or withdrawal reflex thresholds were expressed in grams. These limits assured a more precise determination of mechanical withdrawal threshold in experiments aimed to determine the effect of treatments. An arbitrary cut-off value of 100 g was adopted. Laser treatment started 7 days after injury (performed on days 1; 3; 6; 8; 10; 11; 13; 15; 17; 20; 22) and paw pressure test was conducted on the same days immediately before and 30 min after laser treatment. The data were collected by an observer who was blinded to the protocol.

### Incapacitance test

Weight bearing changes were measured using an incapacitance apparatus (Linton Instrumentation, UK) detecting changes in postural equilibrium after a hind limb injury^43^. Rats were trained to stand on their hind paws in a box with an inclined plane (65° from horizontal). The box was placed above the incapacitance apparatus. This allowed us to independently measure the weight that the animal applied on each hind limb. The value considered for each animal was the mean of 5 consecutive measurements. In the absence of hind limb injury, rats applied an equal weight on both hind limbs, indicating a postural equilibrium, whereas an unequal distribution of the weight on hind limbs indicated a monolateral decreased pain threshold. Data are expressed as the difference between the weight applied on the limb contralateral to the injury and the weight applied on the ipsilateral one^44^. This behavioural measurement was performed on days 1; 3; 6; 8; 10; 11; 13; 15; 17; 20; 22 immediately before and 30 min after laser treatment. The data were collected by an observer who was blinded to the protocol.

### Tissue explants

On day 22, after the behavioural measurements, animals were sacrificed and the ipsilateral sciatic nerves were explanted. As previously reported, the portion containing the ligature was eliminated and a distance of 900 μm proximal to the ligation was chosen as optimal for evaluating the effect of laser treatment^9^. Contralateral nerves were also dissected, and equivalent portions were collected. After fixation in 4% buffered neutral formalin solution, the tissue block was embedded in paraffin, then cut in a microtome to 5 *μ*m thickness and mounted on positively charged slides. The spinal cord of each animal was also collected and frozen in N_2_ for western blot analysis.

### Luxol fast blue staininig

To perform the Luxol Fast Blue (LFB) staining, sections were immersed overnight in 0.1% LFB solution at 56–60 °C. After washing, differentiation was initiated by immersion in 0.05% aqueous lithium carbonate for 15 s followed by multiple immersions in fresh 70% ethanol, until white matter could be distinguished and nuclei decolorized. After washing, sections were immersed in 0.8% periodic acid for 10 min and then rinsed in distilled water. Finally, sections were incubated with Schiff’s reagent for 20 min and rinsed in distilled water for 15 min^45^.

The sections were semiquantified by an arbitrary score starting from 1, mild infiltrate and oedema, up to 10, severe infiltrate and widespread oedema. The morphometric analysis was conducted as previously reported^33^. Sections were analyzed under light microscopy (100 × magnification). At least 6 randomly distributed 20X fields within the transversal section of sciatic nerve were captured for each section. Images were examined using an Olympus BX40 microscope (Olympus, Milan, Italy) and photographed using a digital camera Olympus DP50 (Olympus, Milan, Italy).

### Myelin basic protein (MBP) immunohistochemistry

Slides were deparaffinized with xylene and rehydrated in ascending ethanol. Heat-induced epitope retrieval was performed for 3 min in sodium citrate buffer (10 mM Sodium Citrate, 0.05% Tween 20, pH 6.0). After extensive washing in TBS (PBS + 0.025% Triton X-100), endogenous peroxidase was hindered with 0.3% H_2_O_2_ in 0.3% methanol for 15 min. Sections were then blocked by incubation with Ultra V block (Thermoscientific, Milan, Italy) for 10 min. MBP was detected using a mouse anti-MBP antibody (Chemicon, Milan, Italy) diluted 1:100 at 4 °C overnight. After washing in TBS, sections were treated with an anti-mouse HRP-conjugated secondary antibody (1:1000, Invitrogen, Milan, USA) for 1 h at room temperature. Development with 3-amino-9-ethylcarbazole (AEC) chromogen (BioOptica, Milan, Italy) was performed for 10 min at room temperature following the manufacturer’s instructions. After coverslipping, protein expression was determined by image analysis of the slides on a Zeiss Axioimager microscope (Carl Zeiss; Jena, Germany) at 40 × magnification.

### Western blot of inflammatory markers in spinal cords

Protein extraction from spinal cords started with disruption and homogenization using the TissueLyser II (#85300 Qiagen). Samples were lysed on ice with CelLytic™ MT Cell Lysis Reagent supplemented with 2 mM Na_3_VO_4_ and 1x Protease inhibitor cocktail for mammalian cells (Sigma Aldrich). Tissue lysates were centrifuged at 16000 × g for 20 minutes at 4 °C and the supernatants were then collected. Protein concentration was determined using the BCA protein assay kit (#23227 ThermoFisher Scientific)^46^. Electrophoresis (50 μg of protein/sample) was carried out in 4–12% Bis-Tris Gels (Life Technologies, Carlsbad, CA, USA). Proteins were then blotted onto nitrocellulose membranes, incubated overnight with primary antibodies [anti-EAAT2 (ab41621; dilution 1:1000) and anti-iNOS (ab49999, dilution 1:1000) purchased from Abcam, (Cambridge, UK); anti-COX-2 (AM05213PU-N, dilution 1:1000) was from Origene (Rockville, MD, USA); anti-mPGES-1 (Item No. 160140, dilution 1:200) was from Cayman Chemical (Ann Arbor, MI, USA)] and then detected by enhanced chemiluminescence system (BioRad, Hercules, CA, USA). Results were normalized to those obtained by using an antibody against β-actin (purchased from Merck KGaA, Darmstadt, Germany) diluted 1:10000^47^.

### Statistical analysis

Behavioural measurements were executed on 6 rats per group (Sham, CCI, CCI + laser) performed in two different experimental sets. Measurements were taken in duplicate at least 1 min apart, the responses of both left and right paws were measured. Morphometric and immunohistochemical analyses were performed on 6 rats per group, evaluating four to five different sections of sciatic nerve per animal. Comparisons were carried out using Mann-Whitney nonparametric tests^48^. In all cases, the investigator was blind to the experimental status of each animal. Slides from control and experimental groups were labeled with numbers so that the person performing the image analysis was blinded as to the experimental group. In addition, all images were captured and analyzed by an investigator other than the one who performed measures to avoid possible bias. Results were expressed as mean (S.E.M) with One-Way analysis of variance (ANOVA). A Bonferroni’s significant difference procedure was used as a *post hoc* comparison. Data were analyzed using the “Origin 9.0” software (OriginLab, Northampton, MA, USA). Differences were considered significant at a P < 0.05.
